# Autophagy regulates inflammation in intracerebral hemorrhage: Enemy or friend?

**DOI:** 10.3389/fncel.2022.1036313

**Published:** 2023-01-16

**Authors:** Kaijing Fu, Weilin Xu, Cameron Lenahan, Yong Mo, Jing Wen, Teng Deng, Qianrong Huang, Fangzhou Guo, Ligen Mo, Jun Yan

**Affiliations:** ^1^Department of Neurosurgery, Guangxi Medical University Cancer Hospital, Nanning, China; ^2^Department of Neurosurgery, Second Affiliated Hospital, School of Medicine, Zhejiang University, Hangzhou, China; ^3^Department of Biomedical Sciences, Burrell College of Osteopathic Medicine, Las Cruces, NM, United States; ^4^Department of Rheumatism, First Affiliated Hospital of Guangxi Medical University, Nanning, China

**Keywords:** intracerebral hemorrhage, autophagy, secondary brain injury, neuroinflammatory, NLRP3 inflammasome, microglia, mitophagy

## Abstract

Intracerebral hemorrhage (ICH) is the second-largest stroke subtype and has a high mortality and disability rate. Secondary brain injury (SBI) is delayed after ICH. The main contributors to SBI are inflammation, oxidative stress, and excitotoxicity. Harmful substances from blood and hemolysis, such as hemoglobin, thrombin, and iron, induce SBI. When cells suffer stress, a critical protective mechanism called “autophagy” help to maintain the homeostasis of damaged cells, remove harmful substances or damaged organelles, and recycle them. Autophagy plays a critical role in the pathology of ICH, and its function remains controversial. Several lines of evidence demonstrate a pro-survival role for autophagy in ICH by facilitating the removal of damaged proteins and organelles. However, many studies have found that heme and iron can aggravate SBI by enhancing autophagy. Autophagy and inflammation are essential culprits in the progression of brain injury. It is a fascinating hypothesis that autophagy regulates inflammation in ICH-induced SBI. Autophagy could degrade and clear pro-IL-1β and apoptosis-associated speck-like protein containing a CARD (ASC) to antagonize NLRP3-mediated inflammation. In addition, mitophagy can remove endogenous activators of inflammasomes, such as reactive oxygen species (ROS), inflammatory components, and cytokines, in damaged mitochondria. However, many studies support the idea that autophagy activates microglia and aggravates microglial inflammation *via* the toll-like receptor 4 (TLR4) pathway. In addition, autophagy can promote ICH-induced SBI through inflammasome-dependent NLRP6-mediated inflammation. Moreover, some resident cells in the brain are involved in autophagy in regulating inflammation after ICH. Some compounds or therapeutic targets that regulate inflammation by autophagy may represent promising candidates for the treatment of ICH-induced SBI. In conclusion, the mutual regulation of autophagy and inflammation in ICH is worth exploring. The control of inflammation by autophagy will hopefully prove to be an essential treatment target for ICH.

## 1. Introduction

Intracerebral hemorrhage (ICH), the second most common type of stroke, is characterized by non-traumatic brain parenchymal hemorrhage (Nobleza, 2021). It causes severe neurological dysfunction with high morbidity and mortality rates, accounting for 15% of strokes (Shao et al., 2019; Zhang and Liu, 2020). ICH-induced secondary brain injury (SBI) occurs because blood components and hemolysates from ruptured blood vessels contribute to brain tissue damage and cell death by activating inflammatory reactions, cytotoxicity, and excitatory toxicity (Zhu et al., 2019). At present, although removing hematomas *via* a minimally invasive operation can relieve the mechanical compression of hematomas on peripheral brain tissue, there are still no effective treatments for post-hemorrhage-mediated SBI (Shao et al., 2019).

Autophagy, sometimes called “self-eating,” is a cellular process of stress that is dynamic and complex (Yu et al., 2018). It can maintain the stability of the material and energy and ensure cells’ survival in starvation or other types of stimulations, positively contributing to stroke (Ghavami et al., 2014; Zhang and Liu, 2020). Under normal circumstances, autophagy occurs at a basic level in the brain. It is involved in many physiological activities in most cells, such as cell development and death, immune function decline, and anti-aging mechanism (Wang and Zhang, 2019). However, autophagic activity is significantly enhanced in ICH. In addition, iron, heme, thrombin, and other harmful substances play a role in ICH-induced SBI by regulating autophagy. Neuroinflammation, which plays a critical role in ICH-induced SBI, exacerbates the mass effect by increasing the permeability of the blood-brain barrier (BBB) around the hematoma, leading to cell death. Meanwhile, the cells that die release inflammatory mediators that further aggravate neuroinflammation.

Therefore, reduced neuroinflammation is particularly important for treating ICH (Xiao L. et al., 2020). Is there a relationship between autophagy and inflammation in ICH? It remains a fascinating topic for research that the mechanisms are interrelated but have yet to be fully explained. Obviously, multitarget neuroprotective compounds will be a promising strategy to alleviate ICH-induced SBI. Therefore, in this article, we summarized the fundamental mechanisms of autophagy following ICH and the interrelationship between autophagy and inflammation. We also summarize in detail the potential compounds and therapeutic targets of autophagy in regulating inflammation.

## 2. Pathophysiology of ICH

**FIGURE 1:**
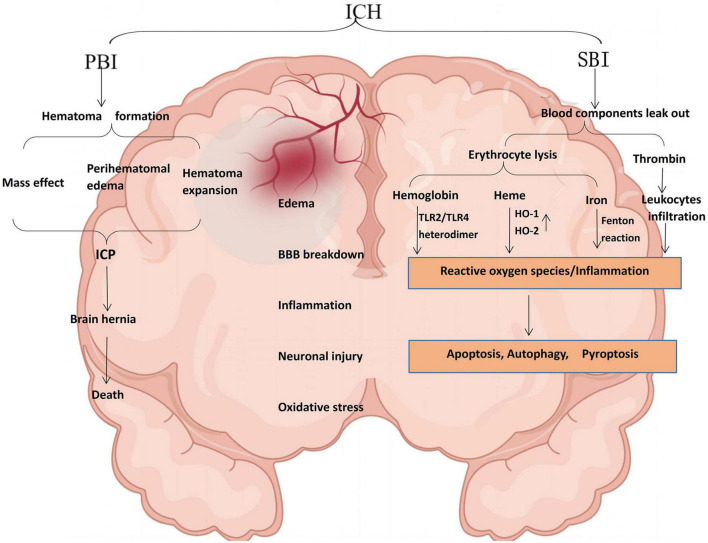
Schematic illustration of pathophysiology after ICH. PBI and SBI are not performed singly, leading to edema, BBB disruption, inflammation, neuronal damage, and oxidative stress. Thrombin can induce significant neuroinflammation in microglia. Most extravasated erythrocytes are trapped in the hematoma and phagocytosed or lysed by microglia/macrophages. In ICH, hemoglobin is a key factor in the inflammation of damaged erythrocyte leakage and is a potent activator of inflammation. Hemoglobin induces proinflammatory cytokine production in microglia through TLR2/TLR4 heterodimer. Heme is partially degraded by HO-1 in macrophages/microglia or HO-2 in neurons to form ferrous iron, carbon monoxide, and biliverdin. The released iron can produce a large amount of ROS through the Fenton reaction and cause oxidative stress damage to nearby tissues. TLR2/TLR4, toll-like receptor 2/4; HO-1, heme-oxygenase-1; ROS, reactive oxygen species (by Figdraw).

Intracerebral hemorrhage usually occurs deep in the brain (basal ganglia and thalamus) (Zhang et al., 2020; O’Carroll et al., 2021), and 30% of patients with ICH may develop hematoma dilation within the first 6 h after ICH (Tschoe et al., 2020). The leading cause is chronically elevated blood pressure (hypertension) and cerebral amyloid angiopathy (Nobleza, 2021). The pathophysiology of ICH is extremely complex, mainly including primary brain injury (PBI) caused by hematoma-related pathological reactions. However, the hematoma is the leading cause of PBI after ICH. It can aggravate SBI, leading to severe neurological dysfunction and even death (Zhang and Liu, 2020). Although these injury pathways are distinct in pathophysiology, the mechanisms by which they injure the brain parenchyma overlap (Magid-Bernstein et al., 2022). For example, inflammation-mediated inflammatory factors and leukocyte infiltration can also lead to BBB breakdown (mainly endothelial cell damage and deformation) after ICH. BBB disruption and increased permeability can also lead to further perihematomal edema, thus forming a vicious circle (Figure 1).

### 2.1. Primary brain injury in ICH

Occurring immediately within the first few days after bleeding, PBI is a direct injury caused by the acute mass effect of an intracerebral hematoma, which causes brain tissue deformation and intracranial pressure. PBI in ICH is characterized by hematoma formation and expansion, the mass effect that leads to greater intracranial pressure (Bautista et al., 2021). The severity of ICH is mainly affected by the site of the hemorrhage and the amount of bleeding. When the blood loss is extensive, the compression of the tissue intensifies. It causes intracranial hypertension (ICP), leading to mechanical damage to adjacent tissue and decreasing cerebral perfusion, eventually bringing severe irreversible neurological dysfunction to the tissue. In addition, mass effects may lead to midline displacement, brain herniation formation, and even death (Magid-Bernstein et al., 2022).

### 2.2. Secondary brain injury in ICH

Previous studies have shown that SBI after ICH is a vital factor leading to neurological dysfunction and a significant determinant of the poor prognosis of patients (Shao et al., 2019). When blood components and damage-associated molecular patterns (DAMPs) are released from necrotic and damaged tissues, injury pathways are activated, including inflammation, blood cytotoxicity, and oxidative stress (Aronowski and Zhao, 2011). SBI after ICH is exceptionally complex, and the most severe consequences of SBI are neuronal cell death or endothelial damage (and subsequent vasogenic edema) (Duris et al., 2018). In addition, hemoglobin, thrombin, heme, and iron cause BBB hyperpermeability after ICH (Bian et al., 2022; Xia et al., 2022).

## 3. Autophagy

There are three types of autophagy, namely, macroautophagy, microautophagy, and chaperone-mediated autophagy. Macroautophagy, also known as autophagy (‘self-eating’) is a major pathway involved in cytoplasmic content recycling and degradation in autophagosomes. Microautophagy can phagocytose cellular solutes into lysosomes and degrade them through the formation of characteristic invaginations of the lysosomal membrane. Unlike macroautophagy, microautophagy does not require the formation of autophagosomes, and its substrates are directly phagocytic and degraded by lysosomes. Chaperone-mediated autophagy is a highly selective pathway recognized only by soluble proteins containing chaperone recognition sites, such as the heat shock protein-70 (HSP70) complex (Kanno et al., 2022). Among the three kinds of autophagy, macroautophagy is the most widely studied and the most characteristic of autophagy. In general terms, “autophagy” refers to macroautophagy. The autophagy mentioned in this review is all macroautophagy (hereinafter, “autophagy”).

### 3.1. Selective autophagy and non-selective autophagy

In mammalian cells, autophagy can also be classified as selective and non-selective (Li et al., 2021d). Selective autophagy is a major process in maintaining cellular homeostasis, in which lysosomes recognize and degrade cargo through the activity of selective autophagy receptors (Lamark and Johansen, 2021; Xu W. et al., 2021). Selective autophagy can be divided into the following categories according to the targeted organelles or particles such as some damaged organelles: mitophagy (impaired mitochondria), reticulophagy (endoplasmic reticulum), xenophagy (pathogens), or aggrephagy (misfolded proteins) (Faruk et al., 2021). Non-selective autophagy is a process in which cytoplasmic components are non-selectively wrapped by bilayer phagosomes to form autophagosomes and transported to lysosomes for degradation and recycling as essential metabolic substrates. It is a physiological reaction to maintain nutrient supplies by stimulating hunger and other behaviors (Li et al., 2021d).

### 3.2. Molecular mechanisms of the canonical autophagy process

**FIGURE 2:**
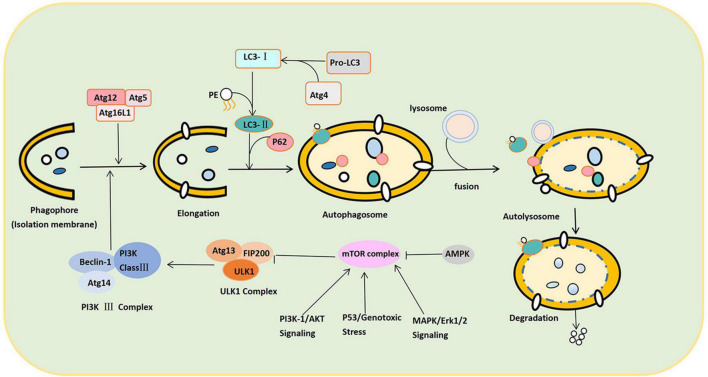
Schematic illustration of the autophagy process. The autophagy process can be divided into the following stages: Induction, nucleation of phagosomes, elongation of phagosomes, completion of autophagosomes, fusion with lysosomes to form autophagosomes, and content degradation. After LC3 binds to phospholipid ethanolamine (PE), LC3-II is anchored to the cell membrane, promoting the expansion and closure of phagosomes and finally forming autophagosomes. P62 links LC3-II to the substrate on the isolation membrane and separates the isolation substrate. In response to SNARE proteins, both ends of the sequestration membrane close and form a complete autophagosome with a bilayer structure. The autophagosome and lysosome fuse to form the autophagolysosome. Under the control of lysosomal-associated membrane proteins, the autophagy outer membrane fuses with the outer lysosomal membrane to form an autophagolysosome. Autophagolysosome degrades the substrate into small molecules such as lipids, nucleosides, and other substances used by the cell.

As shown in Figure 2, the canonical autophagy process involves the sequential and selective recruitment of ATG proteins that can be divided into five sequential steps, including (1) the initiation and nucleation of phagophores; (2) double membrane formation and elongation; (3) the maturation and completion of autophagosomes; (4) lysosomes’ integration with autophagosomes to form autolysosomes; and (5) the degradation of intra-autophagosomal contents by lysosomal enzymes (Yu et al., 2018). In the autophagolysosome, many autophagy-related (ATG) proteins can regulate autophagy, playing an extremely crucial role in the formation of autophagy (Lõrincz and Juhász, 2020). When cells are subjected to various stimuli, such as starvation and energy deprivation, or the activity of the mammalian target of rapamycin (mTOR) complex is inhibited, the initiation of autophagy is mediated by the Unc-51-like autophagy-activating kinase (ULK1) complex, consisting of ULK1/2, Atg13, FIP200, and Atg101 (Jung et al., 2009; Mizushima, 2010; Zachari and Ganley, 2017). Nutritional conditions regulate this complex: When nutrients are plentiful, mTOR phosphorylates ULK1 and Atg13, inactivating the ULK1 complex (Wang and Zhang, 2019). When cells are starved or hypoxic, mTOR activity is inhibited, leading to dephosphorylation and the activation of the ULK1 complex (Fang et al., 2018). Therefore, the ULK1 complex completes the crucial step of inducing autophagy.

Second, the class III phosphatidylinositol 3-kinase (PI3K class III) complex consisting of Beclin-1-Atg14-PI3K class III is an essential component of nucleation and assembly (Wang X. et al., 2020). The PI3K class III complex generates phosphatidylinositol 3-phosphate and recruits additional ATG proteins to form the isolation membrane (also termed the “phagosome”) (Muller et al., 2017). Autophagosome formation and elongation depend on Atg5-Atg12-Atg16L and microtubule-associated protein 1A/1B light chain 3 (LC3) (Yang and Klionsky, 2010). ATG4 can transform Pro-LC3 into LC3. When the complex (ATG12-ATG5-ATG16L) binds LC3 to phosphatidylethanolamine (PE), the cytoplasmic form of LC3 (LC3-I) is anchored to the cell membrane as LC3-II (Stavoe and Holzbaur, 2019). LC3-II promotes the expansion and closure of the phagophore and, eventually, the formation of autophagosomes. Thus, the conversion of LC3-I to LC3-II marks autophagy initiation. After expansion and closure, all the ATGs escape from the autophagosome and return to the cytoplasm. Only the LC3-II is anchored to the autophagosome membrane. Meanwhile, substrate recognition and transport are completed by P62 (Galluzzi and Green, 2019). Hence, P62 can target and bind the substrate and LC3-II, connecting LC3-II with the substrate in the isolation membrane. Once a phagophore is formed, it isolates and coats the substrate (damaged intracellular proteins and organelles). Soluble N-ethylmaleimide-sensitive factor attachment protein receptor (SNARE) proteins transport vesicles and fuse autophagy membranes during autophagy formation. The two ends of the isolation membrane close and form a complete autophagosome with a bilayer membrane structure under the action of SNARE proteins (Tian et al., 2021).

Subsequently, the autophagosome moves along the microtubule system of the cytoskeleton to lysosomes containing cathepsin B and cathepsin D (Hossain et al., 2021). Under the control of lysosomal-associated membrane proteins, the autophagy outer membrane fuses with the outer lysosomal membrane. The inner membrane is degraded by lysosomal enzymes and eventually turns into an autophagolysosome. Hydrolase in the autophagolysosome degrades the substrates into small molecular substances and transports them to the cytoplasm for reuse (Fang et al., 2018).

### 3.3. The regulatory mechanism of autophagy

The regulation process of autophagy can be divided into mTOR-dependent and mTOR-independent pathways. Under normal nutritional conditions, mTOR, which is highly active, recognizes and phosphorylates ULK1 and Atg13 to inactivate the ULK1 complex, which exerts a negative regulatory effect to prevent autophagy (Zachari and Ganley, 2017). In contrast, under starvation or hypoxia, mTOR activity is inhibited. The inhibition of the ULK complex by mTOR is then reduced, which effects the activation of ULK1 kinase and autophagy. The PI3K complex is a downstream regulator of the ULK complex, which can induce an autophagic membrane to turn into nucleation (Shen et al., 2021). There are four main regulatory pathways upstream of mTOR, namely, adenosine monophosphate-activated protein kinase (AMPK), PI3K/protein kinase B (Akt) signaling, MAPK/Erk1/2 signaling, and P53. The inhibition of autophagy occurs through PBK/Akt signaling and MAPK/Erk1/2 signaling, while the activation of autophagy occurs through AMPK and P53 (Xu D. et al., 2021). AMPK can also activate it with the phosphorylation of ULK1, thereby promoting autophagy (Li and Chen, 2019). In addition, p53 is usually present in the cytoplasm. However, when DNA is damaged, it is phosphorylated and translocated to the nucleus. Nonetheless, p53 can affect autophagy in both the cytoplasm and the nucleus (White, 2016). In the cytoplasm, p53 inhibits autophagy by inhibiting AMPK and activating mTOR. It can also directly inhibit FIP200 through a non-mTOR-dependent pathway, inhibiting the autophagy pathway. In the nucleus, though, p53 promotes the transcription of autophagy-related genes to promote autophagy (Tanida et al., 2008; Figure 2).

### 3.4. Molecular mechanisms of ICH-induced autophagy

**FIGURE 3:**
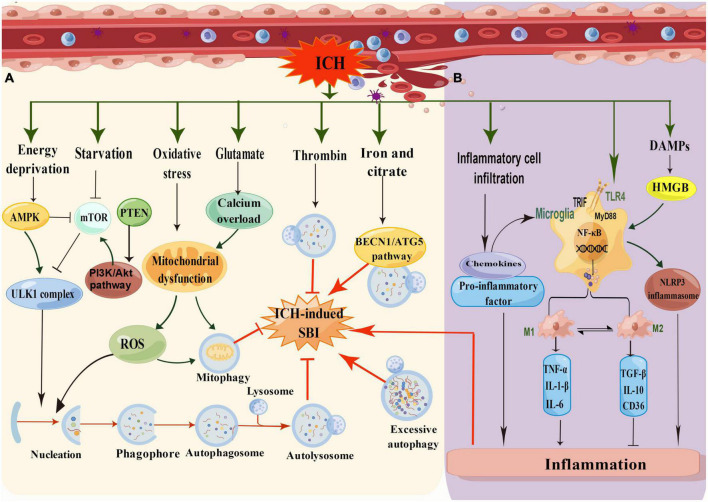
**(A)** Schematic illustration of the mechanism of autophagy in ICH. In low energy states, the increasing ratio of AMP/ATP leads to the activation of AMPK and subsequently inhibits mTOR for induction of autophagy. In addition, the AMPK pathway suppresses mTOR activity, while the cell undergoes induction of starvation or calcium signals. After ICH, oxidative stress produces many ROS, and glutamate causes calcium overload, resulting in mitochondrial damage and inducing mitophagy. Thrombin can reduce SBI by inducing autophagy, whereas iron and citrate can induce autophagy to aggravate SBI through the BECN1/ATG5 pathway. **(B)** Schematic illustration of the mechanism of inflammation in ICH. The inflammatory mechanisms after ICH mainly include microglia activation, inflammatory cell infiltration, toll-like receptor activation, and DMPs mode regulation (by Figdraw).

There is increasing evidence to support the claim that autophagy is activated and involved in the pathophysiology of ICH-induced SBI (Duris et al., 2018). It was found that autophagy was activated 6 h after ICH and peaked at 12 and 24 h (Zhang and Liu, 2020). It has also been revealed that autophagy occurred in the perihematomal area after ICH in rat models, specifically manifested in increased cathepsin D expression and vacuole formation and the conversion of LC3-I to LC3-II. In addition, 24 h after ICH, the protein levels of LC3 and Beclin-1 were increased, and the protein levels of SQSTM1/P62 were decreased, suggesting that autophagy is enhanced in the process of ICH-induced SBI. We have summarized the mechanism of autophagy in ICH (Figure 3A). Nevertheless, this mechanism still merits further exploration.

After ICH, the hematoma expands and presses on the surrounding tissue, leading to edema of the surrounding tissue and resulting in cell hypoxia and starvation. Autophagy is strongly induced by starvation and is an adaptive response to nutrient deficiency and hypoxia, which promotes nutrient recycling (Vargas et al., 2022). In response to a decrease in ATP levels and a rise in AMPK levels, the mTOR pathway was inhibited. Furthermore, energy deprivation, starvation, and other stimuli could initiate autophagy through the ULK1 complex pathway after ICH. In addition, oxidative stress and calcium overload caused by glutamate could lead to mitochondrial dysfunction, producing reactive oxygen species (ROS) to trigger autophagy or mitophagy pathways. It has been mentioned that erythrocyte lysis, iron toxicity, and thrombin play significant roles in ICH-mediated SBI (Zhang and Liu, 2020). Coagulation components or their products, such as thrombin and hemoglobin, participate in endogenous autophagy regulation during ICH. Hematoma-induced thrombin and iron overload accumulation are the leading causes of autophagy.

## 4. Dual role of autophagy in ICH

Many studies suggest that there is no unified theory on the role of autophagy in ICH (Li et al., 2018). The double-sided role of autophagy in ICH may be because different periods and phases of ICH are characterized by different roles in autophagy, mainly depending on the level of autophagy and whether the autophagy flux is complete (Duan et al., 2017).

### 4.1. The role of canonical autophagy in ICH

Studies have confirmed that enhancing autophagy is beneficial for the repair of nervous system diseases (Xu W. et al., 2021). Thrombin is an essential part of the coagulation cascade. A large amount of thrombin is produced to induce hemostasis in the early stage after ICH, which induces leukocyte infiltration and brain edema formation, leading to SBI (Ye et al., 2021). Many studies have found that thrombin is a vital inducer of autophagy after ICH and that autophagy plays a beneficial role in thrombin-induced SBI (Wu et al., 2019). In astrocytes, autophagy was obviously activated 24 h after intracerebral thrombin injection in rats, and autophagy activity reached its peak 3 days after injection. In addition, *in vitro* and *in vivo* data have confirmed the beneficial aspects of autophagy in ICH after SBI induced by thrombin (Shao et al., 2019). The inhibition of thrombin-induced autophagy exacerbated cell death *via* the inhibition of autophagy with 3-MA (3-methyl-adenine), the autophagy inhibitor. Interestingly, ATG7 is an essential autophagy regulator but has no autophagy-unrelated functions. When ATG7 is knocked down using siRNA, the thrombin-induced expression of inflammatory molecules, such as IL-6, is significantly reduced (Shadab et al., 2020). Duan et al. (2016) found that autophagy and ER had distinct effects on ICH at disparate periods. At 6 h after ICH, the autophagy level is too high, and ER stress induction can enhance autophagy, leading to brain injury. However, after 7 days, autophagy may improve the protective effect of ER stress inhibitors by removing the cellular waste produced by early impaired autophagy flux. The primary possible reason is that autophagy flux was damaged 72 h after ICH and restored to integrity after 7 days (Duan et al., 2017). Furthermore, Wang et al. found that the knockdown of the p75 neurotrophin receptor (p75NTR) could promote neuronal autophagy by inactivating the mTOR pathway, presenting protective roles in a rat model of ICH (Wang L. et al., 2020).

The function of autophagy is usually to prevent cellular death. However, excessive autophagy can also cause cell death (Fricker et al., 2018). The excessive elevation of autophagy activity may lead to the accumulation of cellular solutes and the enhanced degradation of essential cellular components in autophagosomes. Excessive autophagy, which blindly hydrolyzes damaged or undamaged cytoplasm and organelles, may lead to increased lysosomal membrane permeability and the release of large amounts of cathepsin into the cytoplasm, thereby aggravating cell damage (Fang et al., 2018). Cathepsin B in lysosomes promotes the release of cytochrome C from mitochondria by regulating the proapoptotic protein Bid, activating caspase family members, and finally inducing apoptosis. Numerous studies have found in rats injected with ferrous citrate and iron that autophagy is highly active and that iron or citrate-induced autophagy has harmful effects on ICH-mediated SBI (Zhang and Liu, 2020). After hemorrhage, iron accumulation subsequently generates abundant ROS, which may induce autophagy and neurotoxicity. However, some studies have found that the inhibition of autophagy can reduce the ICH-mediated SBI induced by ferrous citrate. In addition, heme enhances neuronal autophagy through ER stress and induces cell death through the BECN1 (Beclin1)/ATG5 pathway, and the silencing of Beclin1 and Atg5 or the use of autophagy inhibitors can reduce the release of inflammatory factors in microglia induced by lysed erythrocytes *in vitro*, which is beneficial for alleviating brain injury after ICH (Yang et al., 2020).

There is a great deal of interesting speculation that the beneficial or detrimental effects of autophagy depend in large part on whether the autophagic flux is unimpeded (Li et al., 2018). A complete autophagic flux is essential for homeostasis in almost all cells, especially neurons. According to previous studies, autophagy intensity was primarily determined by the number of autophagosomes or LC3-II. In recent years, it has been found that an increase in autophagosomes is not essentially equivalent to an increase in autophagy levels. An increase in autophagosomes only reflects the induction of autophagy or the inhibition of autophagosome clearance (Li et al., 2018; Zhang and Liu, 2020). From the perspective of autophagic flux, the number of autophagosomes is affected by both formation and clearance. Meanwhile, the heightened activity of cathepsin D and acid phosphatase may be responsible for the greater autophagic flux. Autophagic flux can be demonstrated by the disappearance of the autophagic substrate p62/SQTM1 (Fricker et al., 2018). It is necessary to observe not only autophagosomes but also the entire autophagic flux dynamically to assess autophagy accurately and comprehensively to discern whether the whole autophagic flux process is stable.

### 4.2. Selective autophagy in ICH

Earlier studies classified autophagy as a non-selective process, but recent studies have suggested that the selectivity of targeted cargo is achieved through a series of autophagy receptors (Xu et al., 2015). In 2014, at the Vancouver Symposium on Autophagy, scholars focused on the importance of autophagy as a selective mechanism in pathological conditions, emphasizing the role of autophagy in the strict regulation and precise targeting of substrates (Li et al., 2018). It suggests that a complete understanding of the regulation of selective autophagy in disease may accelerate the development of autophagy-based therapies. Two kinds of selective autophagy, such as mitophagy and ferritinophagy, have been found to play a role in ICH. The roles of selective autophagy in stroke have been reported (Table 1). Currently, most literature on selective autophagy in ICH focuses on mitophagy.

**TABLE 1:** Selective autophagy characteristics and effect in stroke.

Process	Major cargo	Related protein	Type of stroke	Regulatory drugs/Targets	Effect	References
Mitophagy	Damaged or superfluous mitochondria	PINK1/Parkin pathway	ICH	Scalp acupuncture	Enhancing mitophagy and reducing apoptosis.	Li et al., 2021b; Liu P. et al., 2021
			Ischemic stroke	Methylene blue/Rapamycin/DHA	Promoting mitophagy to protects neurons	Li Q. et al., 2014; Di et al., 2015; Sun et al., 2022
		FUNDC1 pathway	ICH	–	Inhibition of NLRP3-mediated inflammation	Zheng et al., 2021b
			Ischemic stroke	tPA/Electroacupuncture pretreatment	Promoting mitophagy to protects neurons	Cai et al., 2021; Tian et al., 2022
		Nrf2/OPTN	ICH	–	Inhibition of NLRP3-mediated inflammation	Cheng et al., 2021
		p53 pathway	ICH	Electroacupuncture at GV20–GB7	Upregulated mitophagy and inhibited apoptotic cell death.	Guan et al., 2021
		BNIP3/LC3	Ischemic stroke	–	Activates excessive mitophagy	Shi et al., 2014
		SIRT3/AMPK	Ischemic stroke	Stilbene glycoside	Promotes mitochondrial autophagy and inhibits apoptosis.	Li et al., 2021e
		AMPK-dependent signaling	SAH	Metformin	Promoting mitophagy to protect neurons	Zhang et al., 2022c
		Keap1/Nrf2/PHB2 pathway	SAH	Mitoquinone	Inhibited oxidative stress-related neuronal death	Zhang et al., 2019
		VDACs/LC3	SAH	VDACs	Protects neurons	Li J. et al., 2014
		NLRP3 inflammasome	SAH	Melatonin	Inhibition of NLRP3-mediated inflammation	Cao et al., 2017
Ferritinophagy	Ferritin	USP14/NCOA4	Ischemic stroke	–	Induce ferroptosis and aggravated brain damage	Li et al., 2021a
		NCOA4/cGAS-STING pathway	Ischemic stroke	–	Aggravates early cerebral ischemia-reperfusion injury	Li B. et al., 2023
		ATG5	SAH	–	Induce neuronal ferroptosis	Liang et al., 2022
		Beclin1/LC3-II/LC3-I	SAH	Baicalin	Suppressed autophagy-dependent ferroptosis	Zheng et al., 2021a
ER-phagy	Damaged or superfluous endoplasmic reticulum	PERK/IRE1 signaling	Ischemic stroke	Melatonin	Attenuate acute neuronal injury	Feng et al., 2017
		PERK signaling/ER stress-autophagy axis	Ischemic stroke	PTP1B inhibitor	Inhibit ER stress-dependent autophagy to neuronal damage and neurologic deficits	Zhu et al., 2021
		ROS/Nrf2/HO-1 signaling pathway	SAH	Hydrogen-rich saline	Attenuate neural autophagy and ER stress.	Jiang et al., 2021

SAH, subarachnoid hemorrhage; tPA, tissue-type plasminogen activator; VDACs, voltage-dependent anion channels; NCOA4, nuclear receptor coactivator 4; USP14, ubiquitin-specific peptidase 14; HO-1, heme oxygenase-1; DHA, docosahexaenoic acid; PTP1B, protein tyrosine phosphatase 1B; PERK, R-like endoplasmic reticulum kinase; IRE1, inositol-requiring enzyme-1.

#### 4.2.1. Mitophagy

Mitophagy is one of the most recognized types of selective autophagy and is the mechanism of the specific autophagic elimination of mitochondria (Lou et al., 2020). Mitophagy, as a mechanism of mitochondrial quality and quantity control, selectively identifies and eliminates dysfunctional or superfluous mitochondria by autophagy, thus playing a role in protecting the brain (Guan et al., 2018). Many mitophagy receptors link target mitochondria to autophagosomes for degradation in response to various environmental or intracellular stresses. It remains to be determined how mitophagy is regulated, whether it is inducted or inhibited, and its specific role in ICH. Mitophagy is restrictively controlled by several proteins, including phosphatase and tensin (PTEN)-induced putative kinase (PINK1) and FUN14 domain containing 1 (FUNDC1) (Li et al., 2021d). The decreased oxygen metabolism in hematomas after ICH leads to mitochondrial dysfunction. In damaged mitochondria, the significant decrease in intracellular ATP and Ca2+ overload and the increase in ROS production are essential factors aggravating the SBI caused by ICH (Guan et al., 2018; Bian et al., 2022). Excessive ROS in tissues or cells produced during oxidative stress can induce autophagy through various mechanisms to prevent further oxidative damage overload in the brain after a hemorrhage (Yao et al., 2021). In addition, PTEN-induced kinase 1 (PINK1) is a mitochondrial-targeted protein kinase. By encouraging mitophagy in microglia, PINK1 defends against ICH-induced SBI, and PINK1 overexpression can treat ICH-induced behavioral problems (Li et al., 2021b). As a vital component of the mitochondrial contact site and cristae junction organizing system, MIC60 plays a significant role in maintaining the mitochondrial structure and functioning. After ICH, mitochondrial damage, mitochondrial crest remodeling and the reduction of MIC60 protein after the PINK1 level decrease, and Parkin localization errors are observed. The overexpression of MIC60 could maintain mitochondrial structural integrity and reverse ICH-induced neuronal cell death and apoptosis (Deng et al., 2021).

#### 4.2.2. Ferritinophagy

As a selective autophagy type, ferritinophagy mediates the degradation of ferritin and the release of free iron and engages in the regulation of intracellular iron content (Vargas et al., 2022). Ferritin chelates free iron and ensures that iron homeostasis is maintained at tolerable levels within cells. However, an overload of ferritin can cause brain damage and cell death (Li et al., 2021a). The release of free iron after erythrocyte lysis and from ferritin stores may influence oxidative stress, glutamine release, and inflammation after hemorrhagic brain injury. Nuclear receptor coactivator 4 (NCOA4) mediates ferritin degradation *via* the autophagosome-lysosome pathway through ferritinophagy (Li B. et al., 2023). With the help of the autophagy proteins ULK112 and FIP200, Tax1-binding protein 1 binds directly to NCOA4 and promotes ferritin lysosomal localization and degradation under iron-depleted conditions (Zheng et al., 2021b).

#### 4.2.3. Reticulophagy (ER-phagy)

As a type of selective autophagy, reticulophagy involves the selective degradation of ER fragments. Reticulophagy is induced by ER stress to maintain the stable state of ER, and intracellular reticulophagy is maintained at a low level under normal physiological conditions. The ER activates reticulophagy when it receives harmful signals, including nutritional deficiencies or unfolding protein aggregation (Wilkinson, 2019). Interestingly, selective degradation of the ER by autophagy has become one of the typical responses to ER stress (Thangaraj et al., 2020).

## 5. ICH-induced inflammation

Neuroinflammation is a crucial factor in ICH-induced SBI and may develop shortly after ICH and peak a few days later (Li et al., 2021c). Inflammation caused by various proinflammatory factors, such as IL-1β, IL-18, and TNF-α, leads to the destruction of the BBB, exacerbated brain edema, and neuronal death (Tschoe et al., 2020; Li H. et al., 2022). There are many reactive inflammatory processes after ICH, including microglia activation, inflammatory cytokine release, and leukocyte infiltration (Figure 3B; Ye et al., 2021). After ICH, the hemolytic components of red blood cells (hemoglobin and iron) and plasma proteins (thrombin and fibrinogen) are released into the brain tissue, leading to inflammatory reactions *via* the activation of the immune, oxidative stress, hemostasis, and other systems (Fu et al., 2021a). After a hemorrhage, neuronal injury and DAMPs trigger proinflammatory responses that increase BBB permeability around the hematoma and worsen the mass effect (Wu et al., 2020a).

### 5.1. Mobilization and activation of inflammatory cells

Neuroinflammation is characterized by the polarization of microglia toward the proinflammatory phenotype and the release of proinflammatory factors (Taylor et al., 2017). Neuroinflammation occurred on day 1 post-ICH, and changes in microglia size and morphology were observed. Activation of microglia leads to the infiltration of macrophages and T cells, which release inflammatory cytokines, chemokines, free radicals, and other potentially toxic chemicals, triggering a cascade of inflammatory responses. Shtaya’s study showed that the accumulation of neutrophils on the endothelial surface is considered a marker of intracerebral microvascular inflammation within 1–4 days after ICH (Shtaya et al., 2019). The proinflammatory factors (TNF-α and IL-1β) and ROS produced by aseptic inflammation after ICH promote the activation of endotheliocytes, which change their phenotype and connexin composition, and express more selectin and adhesion molecules, which is a crucial step in recruiting white blood cell from peripheral blood (Eser et al., 2022). The accumulation of astrocytes in the area surrounding the hematoma was observed 1–3 days after the onset of ICH (Scimemi, 2018). In addition, astrocytes secrete various proinflammatory factors (such as IL-1β and IL-6), which aggravate the neuronal injury and brain injury (Tschoe et al., 2020).

### 5.2. Activation of inflammasome

The inflammasome is a crucial component of innate immunity and is usually composed of the pattern recognition receptor (PRR) containing the pyrin domain, the apoptosis-associated spot-like protein (ASC), and the effector protease caspase-1 (Broz and Dixit, 2016). When stress factors, such as DAMPs, are detected, PRRs divide and activate caspase-1, which leads to the activation of interleukin-1β (IL-1β) and interleukin-18 (IL-18), ultimately resulting in inflammation (Chung et al., 2020). Nod-like receptors (NLRs) are PRRs involved in detecting invading pathogens and initiating innate immune responses. This section mainly describes the role of NLRP inflammasomes in ICH. As the most characteristic inflammasome, the NLRP3 inflammasome plays a crucial role in developing an immune response and disease. NLRP3, pro-caspase-1, and ASC aggregate to form NLRP3 inflammasomes (Huang et al., 2021; Gu et al., 2022). Studies have shown that the NLRP3 inflammasome expression is significantly elevated after ICH (Xiao L. et al., 2020). Ma et al. demonstrated that the NLRP3 inflammasome activation amplifies the inflammatory reaction, promotes neutrophil infiltration, aggravates brain edema, and worsens neurological functioning after ICH in a mouse model of autologous blood injections. There is increasing evidence that the NLRP3 inflammasome is a critical component of the inflammatory reaction in strokes (Gao et al., 2017; Luo et al., 2019). In addition, mature NLRP3 inflammasomes can promote IL-1β and IL-18 maturation and recruit peripheral immune cells, which can amplify an inflammatory reaction (Xiao L. et al., 2020).

The NLRP6 inflammasome is a critical intracellular innate immune sensor shown to regulate immune responses. NLRP6 recruits the adaptor apoptosis-associated ASC and inflammatory caspase-1 or caspase-11 to form an inflammasome (Zheng et al., 2021a). NLRP6 could regulate inflammation in inflammasome-dependent and non-inflammasome-independent ways. The expression of the NLRP6 inflammasome increased in perihematomal brain tissue from 6 h to 3 days and peaked 1 day after ICH. However, the role of NLRP6 in ICH remains controversial (Wang P. F. et al., 2017).

## 6. Autophagy regulates inflammation in ICH

The autophagy mechanism is linked to most cellular stress response pathways, especially inflammatory pathways (Zheng et al., 2021e). Autophagy and inflammation are significant pathologies of ICH-induced SBI; however, their relationship remains unclear (Durocher et al., 2021). Many studies have illustrated that autophagy activation could block inflammation, inhibiting the body’s inflammatory response and reducing the disease in inflammatory tissue injury (Matsuzawa-Ishimoto et al., 2018). Nonetheless, microglial autophagy can also promote inflammation and aggravate SBI. What is the relationship between ICH-induced autophagy and ICH-induced inflammation, and is it an enemy or a friend?

### 6.1. Autophagy inhibits the inflammasome

**FIGURE 4:**
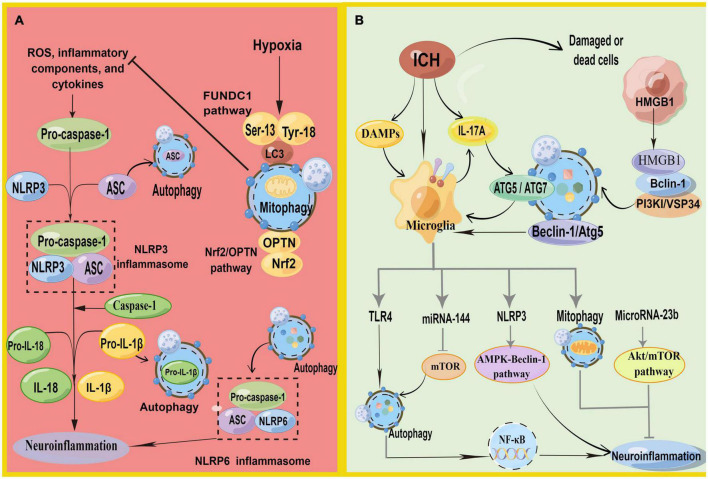
**(A)** Schematic illustration of autophagy regulates NLRP3 inflammasome and NLRP6 inflammasome. NLRP3, pro-caspase-1, and ASC aggregate to form NLRP3 inflammasome. Autophagy antagonizes inflammation mainly in three parts: (1) P62 can recognize ubiquitinated ASC and induce autophagy to degrade inflammasome adaptor protein ASC selectively. (2) Decomposition and removal of pro-IL-1β, the substrate of inflammatory mediators. (3) Can inhibit NLRP3 inflammasome activation by removing endogenous inflammasome activators, such as ROS. Meanwhile, mitophagy antagonizes neuroinflammation-mediated NLRP3 inflammasome *via* FUNDC1 and Nrf2/OPTN pathways. In addition, autophagy promotes inflammasome-dependent NLRP6-mediated inflammation after ICH. **(B)** Schematic illustration of autophagy in microglia regulates autophagy in microglia. HMGB1 combines with Beclin1 and PI3K I/Vsp34 to activate autophagy. Autophagy promotes microglial activation and microglia-mediated inflammation *via* the HMGB1/TLR4/MyD88, Beclin-1-Atg5 pathway. IL-17A-mediated autophagy activation induced microglial activation and microglial inflammation *via* ATG5/ATG7. In addition, microglial autophagy can regulate inflammation through TRL4, miRNA-144, NLRP3, mitophagy, and Microrna-23b (by Figdraw).

Increasing evidence suggests that autophagy inhibits the neuroinflammation mediated by NLRP3 inflammasome activation and, correspondingly, attenuates inflammatory damage (Biasizzo and Kopitar-Jerala, 2020; Figure 4A). Recent studies have depicted that autophagy reduces caspase-1 production by inducing the selective clearance of damaged mitochondria in cells, thereby affecting NLRP3 inflammasome activation and NLRP3-ASC assembly (Plaza-Zabala et al., 2017). Moreover, p62 can recognize ubiquitinated ASC and induce autophagy to degrade inflammasome complexes selectively (Zhao et al., 2021). In addition, when autophagy is impaired, Beclin-1 reduction leads to the enhanced release of IL-1β and IL-18 from the microglia. Autophagy inhibits NLRP3 inflammasome overactivation by removing pro-IL-1β through the autophagic lysosomal pathway (Plaza-Zabala et al., 2017; Houtman et al., 2019). In conclusion, autophagy removes NLRP3 inflammasome activators, NLRP3 inflammasome components, and inflammatory cytokines, reducing inflammasome activation and inflammation (Biasizzo and Kopitar-Jerala, 2020). Additionally, the inflammation caused by ischemic stimulation can be inhibited by autophagy *via* the mTOR and AMPK pathways and by inhibiting inflammasome activation (Mo et al., 2020). However, the mechanism of autophagy regulating inflammation in ICH still necessitates detailed research.

Xiao H. et al. (2020) found that NLRP6 can reduce brain damage by lessening inflammation and inhibiting autophagy after ICH with the NLRP6 gene knockout. They also demonstrated that autophagy promoted inflammasome-dependent NLRP6-mediated inflammation after ICH. However, the role of NLRP6 in ICH remains controversial (Wang P. F. et al., 2017).

### 6.2. Mitophagy inhibits NLRP3 inflammasome

Reactive oxygen species generation is a vital activation pathway of NLRP3. The primary source of ROS has damaged mitochondria. Mitochondria play a vital role in initiating and regulating NLRP3 inflammasomes and are the basis for NLRP3 inflammasome activation. Mitophagy can inhibit NLRP3 inflammasome activation by removing endogenous activators of the inflammasome, including ROS, inflammatory components, and cytokines (Biasizzo and Kopitar-Jerala, 2020; Zhao et al., 2021). The loss of autophagy leads to the accumulation of damaged mitochondria and increased ROS. Currently, mitophagy has been shown to inhibit neuroinflammatory-mediated NLRP3 inflammasome attenuation ICH-induced SBI through the FUNDC1 and nuclear factor E2-related factor 2 (Nrf2)/Optineurin- (OPTN-) mediated autophagy pathways (Cheng et al., 2021; Zheng et al., 2021c). FUNDC1 is a mammalian mitophagy receptor containing LC3-interacting regions capable of interacting with LC3. Hypoxia is one of the most common conditions for inducing the FUNDC1 pathway, and it is a common pathological state in tissues surrounding the ICH. The function of FUNDC1 is regulated by phosphorylation at Ser13, Tyr18, and Ser17. During hypoxia, phosphoglycerate mutase family member 5 phosphatase dephosphorylates Ser13 of FUNDC1, subsequently activating mitophagy by binding to LC3 (Chen et al., 2016; Wu et al., 2017). Zheng et al. found that FUNDC1 protein levels were upregulated and peaked 12 h after ICH (Zheng et al., 2021c). They first demonstrated that FUNDC1 alleviated ICH-induced inflammation by inhibiting NLRP3-mediated inflammation by promoting mitophagy. In addition, the inhibition of FUNDC1-mediated mitophagy markedly increased IL-1β production *in vitro* and *in vivo* (Huang et al., 2020). Optineurin is an autophagy receptor and an autophagy inducer that participates in the initiation of autophagosome membrane formation (Ying and Yue, 2016; Qiu et al., 2022). Cheng et al. found that Nrf2 could interact with the mitophagy receptor OPTN and regulate OPTN-mediated mitotic phagocytosis to effectively remove damaged mitochondria and inhibit NLRP3 inflammasome activation after ICH, thereby alleviating SBI after ICH by collecting human ICH brain flecked for iTRAQ-based quantitative proteomics (Cheng et al., 2021).

### 6.3. Microglial autophagy regulates inflammation

Surprisingly, more evidence suggests that autophagy does not always fight inflammation in ICH but promotes neuroinflammation and exacerbates ICH-induced SBI. The process of autophagy-promoting inflammation is inseparable from microglia (Figure 4B), especially TLR4, on the microglial membrane.

#### 6.3.1. TLR4-mediated autophagy pathway

TLRs belong to a large family of pattern-recognition receptors. TLR4-mediated microglial activation is a pivotal factor in inflammatory injury in ICH (Lee et al., 2019). TLR4 is activated during ICH by several endogenous ligands with injury-related molecular patterns, including hemes and fibrinogen. The activation of TLR4 leads to downstream inflammatory signaling cascades, including the myeloid differentiation primary response 88(MyD88)/TRIF pathway (Gu et al., 2018). TLR4 was upregulated in the early ICH (Deng S. et al., 2022). Interestingly, ROS accumulation in the microglia can trigger the release of inflammatory mediators by activating signaling molecules, including mitogen-activated protein kinase and NF-κB. The activation of microglia and inflammation are associated with TLR4-mediated autophagy. TLR4 synuclein was accompanied by a notable increase in P62, while the levels of other autophagic receptors remained constant. This process is mediated by the activation of NF-κB and promotes the transcription of P62. Increasing evidence demonstrates that the inhibition of TLR4 and its downstream molecules can alleviate the brain injury caused by ICH.

Yang et al. (2015) treated microglia cultured *in vitro* with exogenous heme chloride and observed greater TLR4 expression, as well as proinflammatory cytokines, and proved that autophagy was involved in TLR4-mediated inflammatory damage and the migration of microglia. They also found that 3-MA reduced microglial activation and inflammatory damage caused by lysed red blood cells, thereby augmenting neurological functioning in ICH mice models. This mechanism may occur because autophagy promotes an inflammatory reaction through the high-mobility group box-1 (HMGB1)/TLR4/MyD88 pathway (Lei et al., 2022). HMGB1 has been widely reported to play a central role as a regulator of autophagy and in initiating proinflammatory cytokine production (Xu et al., 2019). After ICH, peripheral nerve cells are damaged, and HMGB1 can be passively released from necrotic or apoptotic cells and transported from the nucleus to the cytoplasm. HMGB1 in cytoplasm mainly promotes the binding of Beclin1 and PI3K I/Vsp34 to activate autophagy by binding to the autophagy factor Beclin1 (Zhang et al., 2022a). Some research has revealed that HMGB1 upregulates the expression of TLR4 and MyD88, causing neurological deficits, and autophagy promotes microglial activation and microglia-mediated inflammation through the HMGB1/TLR4/MyD88 axis in the acute phase of ICH (Lei et al., 2022).

#### 6.3.2. IL-17A pathway

IL-17A is one of the cytokine members of the IL-17 family, which is produced by a subset of T cells. IL-17A promotes the activation of microglia by stimulating the expression of ICH-induced cytokines, including IL-1β and IL-6, TNF-α, as well as downstream signaling molecules, including P65, TRIF, NF-κB, and MyD88. The inhibition of autophagy with RNA interference in essential autophagy genes (ATG5 and ATG7) reduced microglial autophagy and inflammation and ameliorated neurological functioning in a mice model of ICH (Shi et al., 2018). Consequently, IL-17A-mediated autophagy activation induced microglial activation and microglial inflammation.

#### 6.3.3. miRNA-144-mTOR pathway and microRNA-23b-mTOR pathway

miRNAs play a crucial role in inflammation by negatively regulating the expression and function of genes. In addition, miRNA-144 targets mTOR through direct interaction with the 3’-untranslated regions and mutations at the binding site cancel the responsiveness of miRNA-144. Yu et al. detected the autophagy activity, inflammation, and expression of miRNA-144 of microglia in ICH and knocked out endogenous miRNA-144 (Yu et al., 2017). Meanwhile, they demonstrated that miRNA-144 enhanced autophagy activity and the inflammation of microglia after ICH through the mTOR pathway. Furthermore, hemoglobin-mediated autophagy and the inflammation of microglia are mediated by miRNA-144. In addition, microRNA-23b plays an anti-inflammatory role in many diseases and is downregulated in patients with ICH (Wang Z. et al., 2017). MicroRNA-23b negatively regulates inositol polyphosphate multikinase-mediated autophagy, after which it participates in post-ICH anti-inflammatory effects *via* the Akt/mTOR pathway (Hu et al., 2019).

#### 6.3.4. IL-33 pathway and Beclin-1-Atg5 pathway

Interleukin-33 (IL-33) is a new member of the IL-1 family cytokines, and is mainly used as a novel serum prognostic marker in ICH. Its signals through a heterodimer comprising the IL-1 receptor-associated protein ST2L and IL-1RACP to play a biological function is mainly used as a novel serum prognostic marker in ICH (Miao et al., 2021). IL-33 plays a neuroprotective role in inhibiting autophagy and inflammation by increasing P62 and decreasing the expression of LC3-2 and Beclin-1 (Gao et al., 2017). IL-33 also reduces neuronal and white matter damage by promoting the M2-like microglia polarization of microglia after ICH, thereby optimizing neurological outcomes (Chen Z. et al., 2019).

Yuan et al. (2017) found that autophagy promoted microglial activation *via* the Beclin-1-Atg5 pathway in ICH. They also demonstrated that the inhibition of autophagy with pharmacological inhibitors or RNA interference with essential autophagy genes reduced microglial activation and inflammation in ICH and reduced brain damage.

## 7. ICH-induced cell death

The modes of cell death following ICH can be categorized into autophagy, apoptosis, ferroptosis, necrosis, and pyroptosis (Table 2). Autophagy interacts with various forms of cell death, making it difficult to separate the roles of autophagy (Denton and Kumar, 2019). Therefore, the concept of cell death that is solely dependent on autophagy remains somewhat controversial.

**TABLE 2:** Comparison of different ICH-induced cell death.

Types of cell death	Feature	Inducers	Mechanism/Related pathway	Outcome in ICH
Autophagy	As a recycling process, lysosomal mediated catabolism mechanism.	Oxidative stress, inflammation, and accumulation of free iron thrombin	mTOR signaling cascade NF-kB mediated signal pathway. The PI3K/Akt pathway; Mitophagy pathway.	Neuroprotective/nerve cell apoptosis
Apoptosis	DNA fragmentation and apoptotic body	Caspase 3,6,7,8,9 Inflammatory factors	Death receptor-mediated; apoptosis pathway. Mitochondrial apoptosis pathway. Caspase-independent pathways. other signaling pathways.	Phagocytosis
Ferroptosis	The iron-dependent accumulation of toxic lipid reactive oxygen species	Iron-dependent accumulation of lipid peroxidation Iron, extracellular glutamine	Intracellular iron overload. Inhibiting the cystine/glutamate antiporter p53 signaling pathway	Lipid ROS oxidative lipid damage/neuronal cell death
Necroptosis	Cellular swelling, plasma membrane rupture, and subsequent loss of intracellular contents and lysis.	Releasing harmful substances, such as proteolytic oxidizing agents, cytokines, and hemin toxicity	TNF-α/RIPK1/RIPK3/MLKL IL-1R1/RIPK1/RIPK3; CHIP/RIPK1/RIPK3;	Neurological injury
Pyroptosis	Highly inflammatory form of regulated cell death Exclusively mediated by cleaved caspase-1	Caspase-1, gasdermin D NLRP1/3 inflammasome	Classical pathway NLRP3/caspase-1 NLRP1/caspase-1 and NLRC4/caspase-1 pathway; (CCR5/PKA/CREB)/NLRP1 Pathway NK1R/PKCδ signaling pathways. JNK/caspase-1/IL-1ß	Amplifying inflammatory responses, inflammatory necrosis

TRAF6, tumor necrosis factor receptor-associated factor 6; Nrf2, nuclear factor erythroid-2-related factor 2; DRD1, dopamine receptor D1; CCR5, C-C chemokine receptor 5; cAMP-dependent PKA, cyclic adenosine monophosphate-dependent protein kinase A; CREB, cAMP response element binding; NLRP1, nucleotide-binding domain leucine-rich repeat pyrin domain containing 1; RIPK1, receptor-interacting serine/threonine-protein kinase 1; RIPK3, receptor-interacting serine/threonine-protein kinase 3; MLKL, mixed lineage kinase domain-like.

### 7.1. ICH-induced apoptosis

Apoptosis is a type of programmed cell death, which is a series of coordinated and progressive biochemical reactions that lead to the orderly decomposition of cells. The apoptosis of nerve cells exists in the perihematomal region of the brain, a crucial pathological process after ICH (Fricker et al., 2018). The toxic effects of hematoma components and their degradation products, oxidative stress surrounding hematomas, and thrombin release are the principal causes of ICH-induced apoptosis. In general, apoptosis can be divided into intrinsic (mediated by mitochondrial dysfunction or ER stress) and extrinsic (mediated by cell surface receptors, namely, tumor necrosis factor and apoptosis-related factor receptor system) pathways (Bobinger et al., 2018; Wu et al., 2018). The activation of a series of cysteine aspartate proteases is key to apoptosis, especially Caspase 3, a common downstream effect of multiple apoptotic pathways termed the “executive death protease”(Nagata, 2018).

### 7.2. ICH-induced apoptosis and autophagic cell death

Autophagy-dependent cell death is a unique mechanism that occurs independently of apoptosis or necrosis; however, autophagy-induced cell death is often accompanied or triggered by apoptosis (Denton and Kumar, 2019). There is complex crosstalk between ICH-induced apoptosis and autophagic cell death. Bcl-2 is a crucial protein in the regulation of apoptosis. Besides inhibiting apoptosis, Bcl-2 could also inhibit Beclin-1, thus blocking autophagy (Gao et al., 2017). When external pressure or injury destroys normal intracellular homeostasis, the decrease of Beclin-1 binding with Bcl-2 could lead to the induction of autophagy. Autophagy and apoptosis usually occur in the same cell, and studies have shown that autophagy mainly precedes apoptosis. In general, autophagy and apoptosis are mutually inhibitory processes (Maiuri et al., 2007). Autophagy usually inhibits the occurrence of apoptosis, and the caspase protein activated after the occurrence of apoptosis blocks autophagy (Maiuri et al., 2007). For example, an experiment suggested that heme-induced apoptosis was promoted by autophagy *via* the BECN1/ATG5 pathway (Yang et al., 2020).

### 7.3. Other types of cell death

Ferroptosis is an iron-dependent non-apoptotic cell death mode (Ren et al., 2022). Ferroptosis in ICH is closely related to increased lipid peroxidation, disorders of iron metabolism, and glutathione (GSH)-glutathione peroxidase 4 (GPX4) antioxidant systems (Zhang et al., 2018). Necrosis is a caspase-independent and regulated form of cell death. The mass effect of hematoma formation, including thrombin, hemoglobin, and their metabolites, can lead to the necrosis of peripheral nerve cells through the pathophysiological process. Pyroptosis, a programmed cell death mediated by NLRP1/NLRP3 inflammasomes and the caspase family, is an important mechanism of inflammation-induced neuronal cell death in ICH. Pyroptosis is accompanied by cell lysis, which promotes the release of proinflammatory factors, in turn triggering and amplifying inflammatory responses (Gou et al., 2021).

## 8. The cellular autophagy and inflammatory reaction to ICH

Various cells in the central nervous system (CNS), such as microglia, cerebral endothelial cells, and astrocytes, are involved in SBI after ICH. In particular, these cells play an essential role in both inflammation and autophagy, but the functional significance of their reactions is still unclear.

### 8.1. Neurons

Neurons are the most basic structural and functional units of the nervous system that maintain cellular homeostasis *via* strict protein quality control and elimination of abnormal organelles (Zhao et al., 2021). Generally, neurons are divided into three compartments, namely, the soma, the axon, and the dendrites. Neuronal lysosomes and autophagosomes fuse in the soma of the neuron, and most autophagosomes form in the axon (Sedlackova et al., 2020). Interestingly, some studies have found that the production and transport of autophagosomes mainly occur in axons, so axonal transport is crucial for the clearance of damaged mitochondria. Autophagy is especially important in neurons and can directly affect neuronal death. Therefore, neuronal cell lines have been used as models to analyze autophagy (Benito-Cuesta et al., 2017). However, the role of autophagy in neurons is complex, and current research has achieved satisfactory results. Under physiological conditions, autophagy in the neuron is maintained at a low level, but when stimulated, autophagy is enhanced (Stavoe and Holzbaur, 2019).

### 8.2. Microglia

Microglia, as resident immune cells of the CNS, are mainly responsible for clearing damaged tissues and pathogens and reshaping the extracellular matrix (Liu J. et al., 2021). In a normal brain, they are stationary (Eldahshan et al., 2019). Microglia are sensitive to a variety of transcription factors and growth factors, and they activate rapidly by changing morphology and polarization in response to various brain injuries. Activated microglia respond to stimuli to maintain CNS homeostasis by phagocytosing to remove harmful substances. Previously, we have discussed the interaction between microglial autophagy and inflammation after ICH. The regulation of microglia-mediated neuroinflammation by autophagy is not an isolated process but has complex relationships with other pathophysiological processes, such as apoptosis.

### 8.3. Endotheliocytes

Endotheliocytes are actively involved in the complex process of inflammation in the CNS. Endotheliocytes are significant components of the BBB, a critical barrier separating the CNS from peripheral cells. The BBB maintains CNS homeostasis by restricting the entry of inflammatory molecules and peripheral immune cells into the CNS (Abdullahi et al., 2018). A disrupted BBB is a critical feature of neuroinflammation leading to harmful inflammatory cascades (Zheng et al., 2021d). Autophagy plays an indispensable role in vascular diseases as a regulator of endotheliocytes senescence and inflammation. Autophagy has beneficial effects on BBB integrity. For instance, autophagy in endotheliocytes has been shown to target lipid deposition in vascular walls through selective autophagy (lipid phagocytosis). When stimulated by hypoxia and low energy, vascular endothelial growth factor (VEGF) activates autophagy through AMPK-dependent mechanisms, further enhanced by rapamycin treatment, which increases zonula occludens-1 (ZO-1) levels and protects cells from ROS production. However, the role of autophagy in regulating BBB homeostasis remains controversial, as some studies have shown that the activation of autophagy promotes the degradation of BBB components, such as claudin-5 or occludin, in endotheliocytes and capillaries (Yang Z. et al., 2021).

### 8.4. Astrocytes

Astrocytes, known as “support cells,” are the most numerous glial cells in the CNS. The accumulation of astrocytes in the area surrounding the hematoma is observed 1–3 days after the onset of ICH (Scimemi, 2018). Astrocytes exhibit a stronger autophagy response to hypoxia and starvation by activating AMPK-dependent autophagy protection (Murray et al., 2022). In cultured astrocytes, thrombin activates autophagy (enhancing the conversion of LC3-I to LC3-II) in the brain, showing that thrombin plays a role in ICH-induced autophagy. Astrocyte-derived proinflammatory cytokine IL-17A can inhibit autophagy-mediated microglial activation and neuroinflammation, and it decreases the expressions of TNF-α, IL-1β, and P65 (Shi et al., 2018).

### 8.5. Other cells

Oligodendrocytes are the main targets of white matter injury during the stroke (Fu et al., 2021b) and may interfere with autophagy flux through oxidative stress and mitochondrial damage, thereby preventing autophagy from degrading α-synuclein (α-Syn). However, the effect of oligodendrocytes on the pathophysiology of SBI has not been fully revealed (Kang and Yao, 2019). Autophagy not only contributes to T cell homeostasis and affects T cell repertoires and polarization but also contributes to antigen presentation. Platelet hyperactivity is the hallmark of thrombosis, and a study has demonstrated that substantial autophagy is induced (above basal level) by hemostatic agonists, decreasing platelet aggregation (Banerjee et al., 2019; Paul et al., 2019).

## 9. Autophagy and inflammation-related drugs in stroke

Interestingly, since neuroinflammation has been clearly associated with the extent of ICH-induced SBI, the regulation of neuroinflammation by autophagy is a promising therapeutic strategy. Accordingly, it is exciting to search for compounds or multiple targets that modulate inflammation through autophagy in ICH. Further exploration of the molecular mechanisms underlying the neuroprotective effects of these compounds will help us understand the pathology of ICH and illuminate potential treatments for ICH. In this section, we focus on targets and compounds related to the autophagy regulation of the inflammation treatment of ICH.

### 9.1. Potential targets related to autophagy and inflammation in ICH

Several targets modulate inflammation through autophagy in the ICH (Table 3). MCC950, an NLRP3 inflammasome-specific inhibitor, inhibits NLRP3 derived from microglia *via* the AMPK/Beclin-1 pathway to prevent excessive autophagy and reduce the release of IL-1β into the cell (Zhang et al., 2022b). Besides, alpha 7 nicotinic receptor (α-7nAChR) is a subtype of acetylcholine receptor primarily found in the CNS, and α-7nAChR is activated against inflammatory diseases by triggering cholinergic anti-inflammatory pathways. PNU-282987, an α7-nAChR agonist, activates α7-NachR to reduce inflammatory factors, promote the polarization of macrophages into anti-inflammatory subtypes, repair BBB injury, alleviate acute brain edema, and then recover neurological dysfunction *via* the AMPK-mTOR-P70S6K-associated autophagy pathway (Su et al., 2022). Protocatechuic acid (PCA) is an important phenolic acid in plants with antibacterial, anti-tumor, and anti-oxidation effects. PCA is inhibited mTOR signaling and therefore improves M1/M2 polarization switching and attenuated neuroinflammation by suppressing the activation of the microglia (Xi et al., 2021).

**TABLE 3:** Summary of potential targets related to autophagy and inflammation for ICH.

Effects of autophagy	Targets	Mechanism/Related pathway	Effects	Treatment	References
Anti-inflammatory	FUNDC1	FUNDC1 pathway/NLRP3 inflammasome	Promotes mitophagy attenuates NLRP3-mediated inflammation.	–	Zheng et al., 2021d
	α7nAChR	AMPK-mTOR-p70S6K-associated autophagy	Reduced inflammatory factors, promoted the polarization of macrophage/microglia, repaired BBB injury.	PNU-282987	Hijioka et al., 2012; Su et al., 2022
	mTOR	mTOR pathway	Improves M1/M2 switch and attenuated neuroinflammation.	PCA	Xi et al., 2021
Pro-inflammatory	NLRP3	AMPK/Beclin-1 pathway	Significantly reducing edema formation and improved cognitive dysfunction.	MCC950	Zhang et al., 2022b
	IL-17A	ATG5/ATG7	Contribute to the expressions of TNF-α, IL-1β, IL-6, MyD88, TRIF, NF-κB.	IL-17A-neutralizing antibody	Yu et al., 2016; Shi et al., 2018
	IL-33	LC3-II/Beclin-1	Negatively regulating inflammation and autophagy activation and reducing brain injury and neurological dysfunction induced.	sST2	Gao et al., 2017
	H2S/Cystathionine β-synthase	P2X7R/NLRP3 inflammasome	Inducing endogenous H2S production, inhibiting of P2X7 receptor and attenuating NLRP3 inflammasome mediated neuroinflammation	NaHS	Zhao et al., 2017; Shan et al., 2019
	MicroRNA-23b/IPMK	Akt/mTOR signal pathway	Negatively regulating the targeting of mediated autophagy and inhibiting neuroinflammation.	–	Hu et al., 2019
	miRNA-144	mTOR pathway	Attenuates autophagy-mediated microglial inflammation	–	Yu et al., 2017
	HMGB1	HMGB1/TLR4/MyD88 axis	Contributes to microglial activation and inflammatory injury.	–	Lei et al., 2022; Su et al., 2022
	NLRP6	ASC and caspase-1	Contributing to inflammation and SBI following ICH by activating autophagy	–	Wang P. F. et al., 2017; Xiao H. et al., 2020

IPMK, inositol polyphosphate polykinase; P2×7R, P2×7 receptor; PCA, protocatechuic acid; α7nAChR, alpha-7 nicotinic acetylcholine receptor.

### 9.2. Autophagy-related compounds in ICH and clinical trials

There are several poststroke autophagy drugs in clinical trials (Table 4). Although many autophagy-related drugs have been preclinically tested in the treatment of ICH, few have been tested for neuroprotective clinical efficacy.^1^ Only patients taking minocycline achieved beneficial results. Minocycline is a tetracycline class of oral antibiotics. After the injection of minocycline in rat models, Wu et al. found that Beclin-1 and LC3 II/I were suppressed and that minocycline could reduce brain injury *via* the anti-autophagy and anti-apoptosis pathways (Wu et al., 2016). Minocycline can reduce the iron overload caused by ICH, upregulate the expression of Bcl-2 protein, and restrict the flow of calcium ions into the mitochondrial membrane, thus inhibiting autophagy and heightening neural functioning. Statins such as lovastatin have been shown to reduce the risk of ICH, maintain the integrity of the BBB, and enhance neurological function (Chen C. J. et al., 2019). Hepcidin (a hepatic peptide hormone to regulate iron absorption) and hesperidin (a flavanone glycoside with a wide range of biological effects) could inhibit autophagy and reduce brain injury. However, hirudin (a thrombin inhibitor), luteolin (a group of flavonoids), and acupuncture could activate autophagy for neuroprotective effects.

**TABLE 4:** Autophagy-related compounds in ICH and these compounds or treatment in clinical trials for stroke reported (https://www.clinicaltrials.gov/).

Compound	Autophagy	Molecular mechanism	References	Intervention/Treatment	ClinicalTrials.gov identifier	Disease	Study results
Minocycline	Inhibition	Anti-autophagy and anti-apoptosis pathways	Wu et al., 2016	Minocycline	NCT00630396	Stroke, acute	Has results
					NCT01805895	ICH	Has results
Lovastatin	Inhibition	Inhibited AMPK/mTOR signaling pathway	Deng X. et al., 2022	Lovastatin	NCT00243880	Stroke	No results available
Hirudin	Activation	Upregulation of LC3-I to LC3-II conversion and cathepsin D levels	Hu et al., 2011	Hirudin plus aspirin	NCT02181361	Cardioembolic stroke	No results available
Acupuncture	Activation	mTOR pathway	Liu et al., 2022	Acupuncture	NCT01037894	Hemorrhagic stroke	No results available
					NCT02612441/NCT02210988/NCT02472613/etc.	Ischemic stroke	No results available
					NCT02690493	Cerebrovascular stroke	No results available
					NCT04283591	Post-stroke depression Anxiety disorders	No results available
Luteolin	Activation	Activation of the p62/Keap1/Nrf2 Pathway	Tan et al., 2019	–	–	–	–
Hepcidin	Inhibition	Inhibited LC3-II/LC3-I conversion ratio reduce brain iron	Yang G. et al., 2021; Zhang et al., 2021	–	–	–	–
Hesperadin	Inhibition	Inhibiting the MST4/AKT signaling pathway	Wu et al., 2020b	–	–	–	–
Electroacupuncture at GV20-GB7	Activation	Regulating the balance between mitophagy and apoptosis of p53 pathways	Guan et al., 2021	–	–	–	–

GV20, Baihui; GB7, Qubin; Keap1, Kelch-like ECH-associated protein 1; MST4, ste20-like kinase 4.

## 10. Conclusion

Intracerebral hemorrhage remains a worldwide concern and a serious risk to human longevity. However, the pathophysiology of ICH-induced SBI is extremely complex. Many studies have demonstrated that neuroinflammation and autophagy are promising targets for the treatment of strokes. Damaged organelles and misfolded proteins are degraded during autophagy, a cellular self-degradation process. However, due to the diversity of potential factors affecting autophagy, autophagy does not always act as an enemy against ICH-induced SBI. In contrast, autophagy sometimes contributes to inflammation as a friend of SBI. Is that because the activation of autophagy in ICH is excessive or the flow of autophagy is blocked? To better understand inflammation and autophagy in ICH, we consider it advisable to start with intracranial cells, such as microglia and neurons, although the current research on these aspects is not sufficient. The regulation of neuroinflammation by autophagy to treat ICH is an exciting area of research with many unanswered questions.

## Author contributions

KF, WX, and JY designed the structure of the manuscript. CL, YM, TD, JW, QH, and FG managed the literature searches and analyses. KF wrote the manuscript. JY and LM assisted with the improvement of the manuscript. All authors contributed to the article and approved the submitted version.

## Footnotes

1
https://www.clinicaltrials.gov/
